# Socioeconomic Disparities in Urban Forest Diversity and Structure in Green Areas of Santiago de Chile

**DOI:** 10.3390/plants13131841

**Published:** 2024-07-04

**Authors:** Brian R. Guevara, Sandra V. Uribe, Carmen L. de la Maza, Nélida R. Villaseñor

**Affiliations:** Grupo de Ecología, Naturaleza y Sociedad, Departamento de Gestión Forestal y su Medio Ambiente, Facultad de Ciencias Forestales y de la Conservación de la Naturaleza, Universidad de Chile, Santiago 8820808, Chile; brian.guevara@ug.uchile.cl (B.R.G.);

**Keywords:** biodiversity, environmental justice, Latin America, native species, Santiago de Chile, socioeconomics, urban ecology, urban parks

## Abstract

Urban trees enhance biodiversity, provide ecosystem services, and improve quality of life in cities. Despite their benefits, trees are not distributed equitably, and many cities exhibit a “luxury effect”. Given the importance of public green space for providing access to urban tree benefits, we investigated the relationship between socioeconomic level and tree diversity and structure in 60 green areas in Santiago de Chile. Species richness and total tree abundance did not significantly vary among socioeconomic levels; however, a differential effect was found according to species origin. Introduced tree species exhibited similar abundance and species richness across socioeconomic levels, but native tree species were more abundant and richer in higher socioeconomic level areas compared to lower ones. Tree cover was higher in the high and medium socioeconomic level areas than in the low socioeconomic level area. A higher average DBH was found in the medium socioeconomic level area, which may be explained by older neighborhoods and a legacy of the luxury effect. Our findings reveal that socioeconomic groups are associated with differences in tree cover, width, and the number of native species in public green areas. Consequently, urban residents have different provisions of ecosystem services and opportunities to interact with natural heritage. Increasing the amount of tree cover and native species available to vulnerable groups will reduce disparities.

## 1. Introduction

Biodiversity in cities provides multiple ecosystem services and allows city dwellers to appreciate nature daily [1]. Plant cover in the city promotes physical activity, recreation and social gatherings, and gives more value to neighborhoods, improving people’s quality of life [2,3]. Urban tree cover increases air quality, contributes to carbon sequestration, improves flood resilience, and provides shade, reducing the heat island effect [4,5,6]. In addition, urban trees increase biodiversity in the city by improving habitat quality for native fauna [7,8,9,10]. This high biodiversity in green spaces contributes not only to nature but also to improving the moods and psychological wellbeing of people [11,12,13,14].

Despite the benefits provided by plant cover and diversity, vegetation is not evenly distributed within cities [15]. For example, a positive relationship has been described between plant diversity and the socioeconomic status of people, named the “luxury effect” [15]. Studies in different cities around the world show that a higher household economic income is associated with higher plant cover and access to ecosystem services [16,17,18]. It is common for zones where people of higher socioeconomic status live to have more resources available for green space management that increases the extent of green areas and promotes the planting of species that are not naturally found in the local environment, increasing species richness [15,19].

Tree cover has also been found to be positively related to socioeconomic level in urban areas [18]. Although tree cover can be associated with a higher abundance of trees, this is not always the case. Older trees usually reach greater dimensions than younger ones, which results in higher tree cover in areas where older trees are present [20]. The presence of old trees in urban areas depends strongly on tree management as their persistence is associated with their long-term protection and care, and this is subject to the availability of monetary resources [21,22].

The manifestation of the luxury effect would also depend on the background ecosystem where a city develops. A strong relationship between economic wealth and biodiversity exists in regions with water limitations, where the main factor driving biodiversity differences is irrigation due to water being a limiting variable for vegetation growth [23,24,25]. In cities located in Mediterranean and semi-arid climates, where vegetation is limited and there are higher urban heat effects, increased water availability leads to higher biodiversity [25]. As a result of climate change these low precipitation areas will become drier [26], which could intensify the luxury effect in Mediterranean and semi-arid areas in the future.

If the main driver of the luxury effect is the difference in vegetation management (e.g., planting, maintenance, irrigation) caused by socioeconomic differences [27], it would be expected that the luxury effect would not be significant in public green areas such as parks and squares where management is carried out by the same entity. For example, in Arizona, USA, the authorities base their decisions on city-level strategies, and thus, the management of green areas tends to be similar across areas dependent on the same municipality [28]. However, in large and mega cities, public green areas are often managed by different municipalities or districts that have different resources for their management [16,29,30]. This difference in resource availability might be stronger in capitalist countries than in socialist countries, accentuating differences in species diversity [18].

Here, we investigate whether a luxury effect can be found in public green areas. We focused on Santiago de Chile, a Latin American capital city that exhibits strong social and environmental segregation [31,32]. Thus, we aimed to explore the relationship between socioeconomic level and tree diversity and structure in green areas. We posed the following research questions: (1) Does tree diversity vary across green areas located in neighborhoods within different socioeconomic levels? Does a differential effect exist according to the origin of the species (native and non-native species)? Do tree cover and tree age approximation revealed by diameter at breast height (DBH) change with socioeconomic level? In Santiago de Chile, trees are more diverse where people of higher socioeconomic status live [33,34], and while this relationship was found for the entire city (including public and private properties in different land use areas), we do not know whether this relationship can be found for trees in public green areas. Therefore, our study will contribute to furthering our understanding of disparities in cities and will help public policy makers to consider this and its implications for people’s lives in cities.

## 2. Results

**Figure 1:**
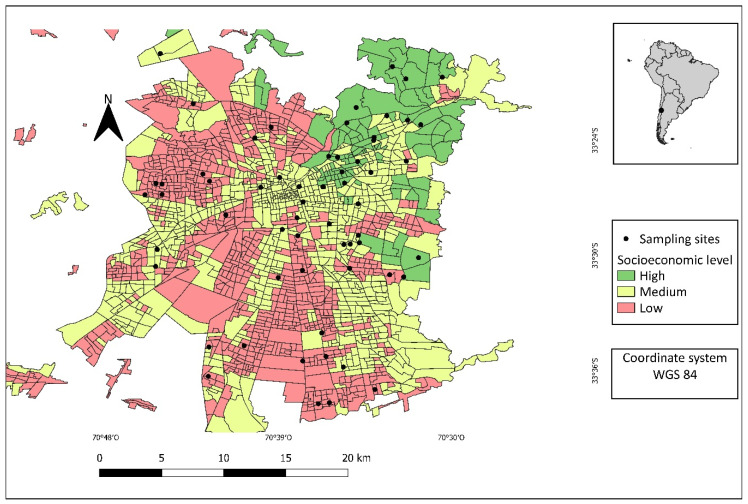
Sampling sites and socioeconomic levels by census blocks in the city of Santiago de Chile.

A total of 117 plots corresponding to 60 urban green areas were sampled in Santiago de Chile (Figure 1). In total, 463 trees corresponding to 70 different species were recorded (Appendix A). Of the 463 trees, 322 individuals (69.5%) corresponded to introduced species and 141 individuals (30.5%) to native species (Table 1). Of the 70 different tree species recorded, 60 (85.7%) were introduced and 10 (14.3%) were native. Two native species were the most abundant species, comprising 20.8% of the total: *Vachellia caven* (Molina) Seigler and Ebinger exhibited a relative abundance of 10.6%, *and Quillaja saponaria* Molina a relative abundance of 10.2% (Appendix A). We recorded (mean ± EE) 3.96 (±3.04) trees per plot, 1.21 (±2.05) native trees per plot, and 2.75 (±2.48) introduced trees per plot (Table 1).

When considering the set of trees recorded in each socioeconomic level and dividing it by the area sampled by socioeconomic level (in ha), green areas in the high socioeconomic level presented a higher abundance of total trees and native trees per hectare sampled (120.85 trees/ha sampled and 49.32 native trees/ha sampled, respectively, Table 2).

**Figure 2:**
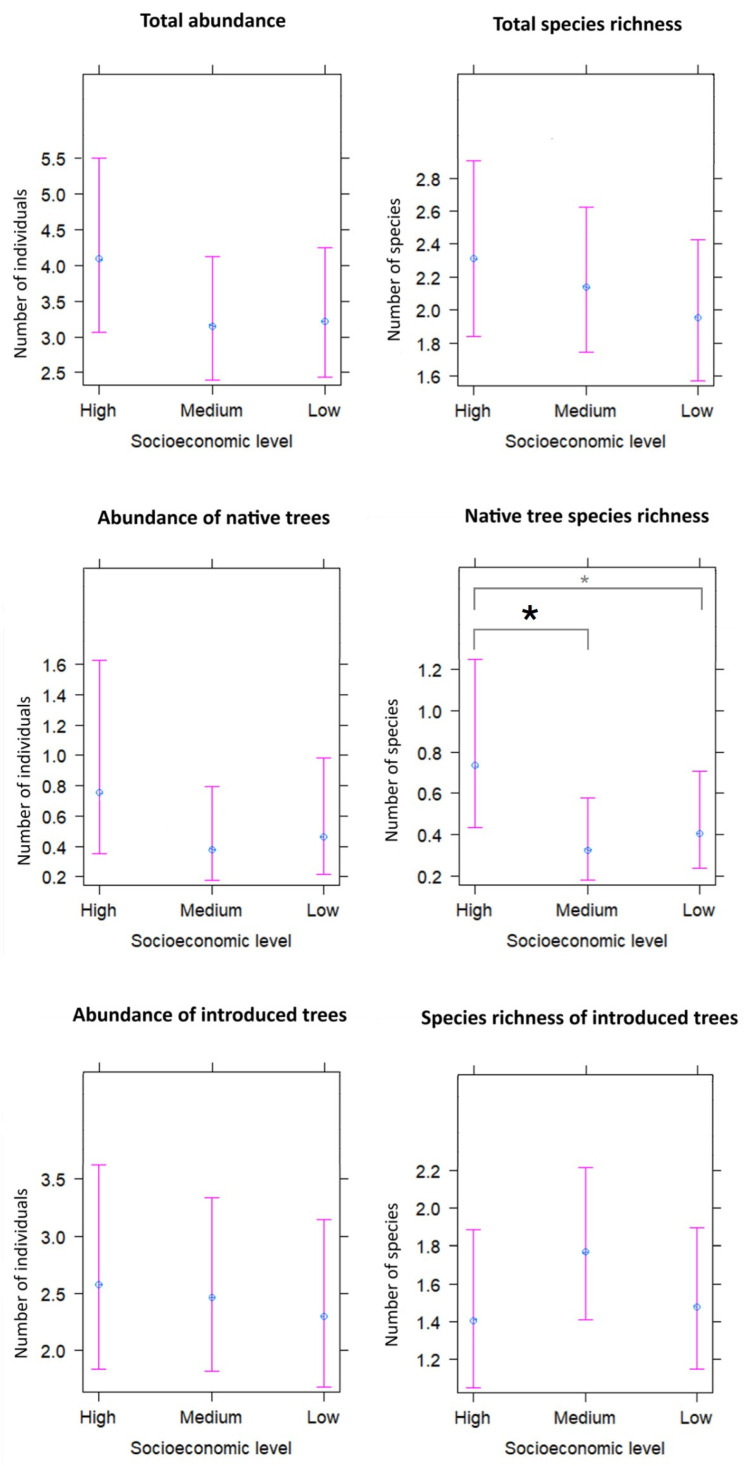
Prediction of the abundance and species richness of total, native, and introduced trees per plot in green areas within different socioeconomic levels in the city of Santiago de Chile according to the Generalized Linear Mixed Models. Gray asterisk shows close to significant differences (*p* < 0.1) and black asterisk shows that statistically significant differences were found (*p* < 0.05).

Although the total abundance of trees and species richness per plot exhibited higher values in the high socioeconomic level, no statistically significant differences were found between socioeconomic levels (*p* > 0.05; Table 3; Figure 2). Native trees were more abundant in the high socioeconomic level (Figure 2); however, the difference between socioeconomic levels was not statistically significant (Table 3). A higher native tree species richness per plot was found in green areas within high socioeconomic level areas compared to the medium socioeconomic level (*p* = 0.03) and low socioeconomic level areas, although in the latter case it was close to significant (*p* = 0.09; Table 3; Figure 2). The abundance of introduced trees per plot was similar among socioeconomic groups and the richness of introduced species per plot was slightly higher in the medium socioeconomic level (Figure 2); however, the difference was not statistically significant (Table 3).

**Figure 3:**
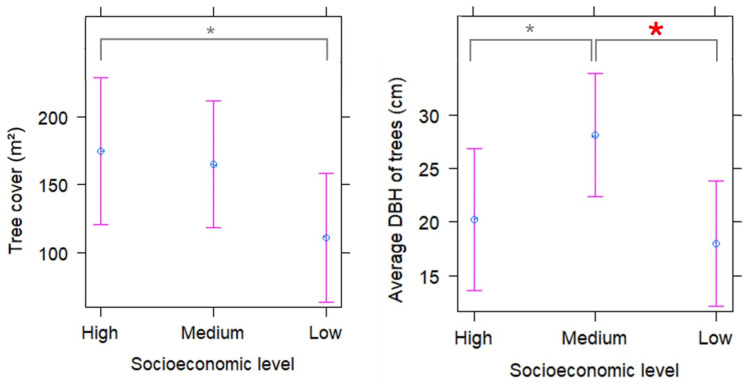
Prediction of tree cover (m^2^) and average tree DBH (cm) per plot in green areas in different socioeconomic level areas in the city of Santiago de Chile according to the Linear Mixed Models. Dots show the estimated mean and bars show the 95% confidence intervals. Gray asterisks show close to significant differences (*p* < 0.1) from Linear Mixed Models and red asterisk shows statistically significant differences (*p* < 0.05) found from Tukey’s test.

Tree cover per plot was lower in green areas in the low socioeconomic level area compared to the high socioeconomic level area, and the difference was close to significant (Table 4; Figure 3). The average DBH per plot was low in green areas in high and low socioeconomic level areas, and it reached a higher value in green areas located in the medium socioeconomic level area (Table 4; Figure 3). Given that in this case the plot suggests differences in the average DBH between medium and low socioeconomic level areas (Figure 3), we performed Tukey’s tests for pairwise mean comparisons and found that the average DBH was significantly higher in plots located in green areas in medium socioeconomic level area than those in low socioeconomic level areas (*p* = 0.04).

## 3. Discussion

In urban green areas, trees are key habitat elements that provide multiple benefits to people. The present study evaluated the relationship between socioeconomic level and the diversity, abundance, cover, and age approximation (DBH average) of trees in green areas in Santiago city. The abundance and richness of tree species did not significantly vary among green areas within different socioeconomic levels, but a differential effect was found according to the origin of the species. While the richness and abundance of introduced trees did not differ significantly between socioeconomic levels, the richness of native species was higher in plots located in green areas in high socioeconomic level areas. In addition, there were differences in tree cover and tree DBH among the socioeconomic levels. The most relevant results are discussed below.

### 3.1. Luxury Effect: Abundance and Tree Diversity

Although the total abundance of trees and species richness per plot exhibited higher mean values in the high socioeconomic level areas, there were no statistically significant differences between the socioeconomic levels. Similar findings showing a lack of a luxury effect have been found in cities where green areas are managed by the same municipality [35]. In the case of Santiago city, green areas are managed by different municipalities with different budgets [29]; however, governmental entities contribute to green space management and have possibly helped to achieve higher equity in terms of tree abundance and species richness. This lack of significant differences among green areas differs from previous research conducted in public and private areas across the city, where municipalities and neighborhoods of a higher socioeconomic level have a higher tree diversity [33,34]. Given that those previous studies considered both private and public areas, they included citizens’ small-scale actions (from individuals and/or families) that reflected economic differences among groups.

### 3.2. Differential Effect According to the Origin of the Species

The luxury effect changed with species provenance. The abundance and diversity of introduced trees in green areas was similar among socioeconomic groups revealing an absence of luxury effect at this scale and agreeing with findings from the Santiago urban forest regarding introduced plants [34]. In contrast, the abundance and diversity of native trees was higher in green areas located in high socioeconomic zones, although statistically significant differences were only found for native species richness [33,34]. In the Santiago urban forest, native tree species are more diverse and abundant in high socioeconomic areas than in low socioeconomic areas. The presence of more native tree species in areas where people of high socioeconomic level live might be due to a higher frequency of remnants of natural or semi-natural vegetation in high socioeconomic zones, which favors the presence of native tree species.

### 3.3. Tree Cover and DBH Averages

Higher tree cover was found in green areas located in zones of high and medium socioeconomic level than those in zones with a low socioeconomic level. This difference could be due to the quality of tree management (e.g., pruning, irrigation, pest control, and/or fertilizers) which depends on the resources available in each municipality [29]. In fact, tree care exhibits a high level of inequity in our city, with trees in poorer zones being subject to pruning of very low quality, largely reducing crown size and damaging tree structure and health [36].

The higher amount of canopy cover in high and medium socioeconomic green areas would provide a greater amount of ecosystem services to users. For example, a higher canopy cover is associated with a larger amount of shade, and thus, a reduction in ambient temperature during the summer season [6]. In addition, a higher amount of canopy cover captures more particulate matter, contributing to improved air quality and, consequently, decreasing respiratory diseases and improving people’s quality of life [37]. It is important to consider that urban air pollutants can also harm trees, and thus managers and planners should carefully select species that can thrive in the urban environment [38].

Trees tended to be wider (higher average DBH) in medium socioeconomic level areas. The oldest neighborhoods in Santiago are in sectors where the medium socioeconomic level is preponderant, for example, in the municipalities of central Santiago, Providencia, and Ñuñoa, so their green areas have older and larger trees. In the past, these areas had a concentration of high-income residents that later moved towards the northeastern part of Santiago [39], thus our result possibly reflects the legacy of a past luxury effect. In addition, there was a trend of there being more introduced tree species in the medium socioeconomic level area, which due to their rapid growth would result in a larger average DBH. Previous research has found that the richness of introduced plants increases with park age, because in older parks long-lived ornamental species were planted according to contemporary landscaping trends [40,41]. Therefore, our study also suggests a positive relationship between neighborhood age, tree age, and introduced tree species richness.

### 3.4. Urban Tree Composition

Tree species composition in green areas is mostly of introduced origin, reaching 69.5% of the individuals sampled. This percentage is similar to previous studies in urban parks managed by the government (e.g., 71% [42]), and lower than the percentage recorded for the Santiago urban forest (e.g., 96% [34] and 86% [34]). The tendency to plant mainly introduced species is due to historical and ornamental criteria implemented in South America after European colonization [43], which has been perpetuated by a higher provision of introduced plants in nurseries [44]. It is expected that the contribution of native species will increase with time, as native tree planting programs are established and promoted at both the city and national level [34,45].

Although introduced species were more numerous, two native species reached the highest abundances in green areas. The most abundant species was *Vachellia caven* (10.6% of total), although it was only recorded in five green areas (out of a total of 60 green areas). This high abundance achieved with a low occurrence in green areas was because they mainly comprised remnant trees from previous natural areas. The second most abundant species was *Quillaja saponaria* (10.2% of total). Unlike *Vachellia caven, Quillaja saponaria* was recorded in 21 green areas, indicating a predilection for its use in urban green areas probably due to its plasticity, drought resistance, climate adaptability [46], and high nursery production [47]. On the other hand, the most abundant introduced species was *Liquidambar styraciflua* L. (7.6% of total), which was also the most frequent introduced species in green areas (13 green areas). The use of this species is mainly ornamental and aesthetic, ignoring its high-water requirement [42].

### 3.5. Recommendations for Green Areas

A common recommendation in the international literature is to promote a high abundance and diversity of trees in green areas, as this would increase the provision of ecosystem services and improve the life quality of people in cities [2,3]. In our city, afforestation and management programs should place special emphasis on increasing tree cover in green areas located in the low socioeconomic level areas, since this vulnerable group has the lowest tree cover that leads to an inequity in the associated benefits.

In order to increase the number of native trees in Santiago’s green areas, it is necessary that government entities invest in planting species with low water requirements, that are resistant to urban pollutants, and that can tolerate current and future high temperatures, favoring native species adapted to the climate [38,48]. Special focus is required for green areas in low and medium socioeconomic level areas, which have a lower richness of native species. For this, the availability and diversity of native species and native plants with conservation problems in nurseries should increase. If possible, each municipality should have its own nursery program that is associated with long-term planning for its green areas and engaging with the local community. The criteria for selecting native species should consider the ecological requirements of the species, and the sustainability, feasibility, and the provision of ecosystem services (and a reduction in ecosystem disservices), not only cultural aspects such as scenic beauty [42]. On the other hand, it is advisable to avoid native species that generate allergic reactions (e.g., *Lithraea caustica* (Molina) Hook. and Arn. [49]) as well as thorny species (e.g., *Vachellia caven*) near children’s playgrounds to avoid damage or injury.

Tree management should be carried out by trained personnel (public or private) so that their interventions do not harm the aesthetics, stability, and health of the trees [36,50]. Finally, equitable access to the ecosystem services provided by trees in green areas should be ensured, regardless of age, gender, ethnicity, or socioeconomic level of the people living in the city.

## 4. Materials and Methods

### 4.1. Study Area

The study area is located in the capital city of Santiago de Chile (33°26′ S and 70°39′ W) (Figure 1). This city has an area of ca. 641 km^2^ and more than 6 million inhabitants distributed in 32 municipalities, being the most populated city in Chile [51]. The climate is Mediterranean according to the Köppen–Geiger classification, with hot and dry summers (December–March) and cool and rainy winters (June–August) [35]. The monthly average temperature for the warmest month (January) is ca. 20 °C, and for the coldest month it is ca. 8 °C (July), while the average precipitation is approximately 312.5 mm per year [52]. However, since 2010, the region has been affected by a prolonged drought with rainfall deficits close to 35% [53].

In this city, landscaping using introduced ornamental species has been a widespread practice and 86% of the trees correspond to introduced species [34]. At the city level, the tree stock is more diverse and a higher diversity of native species is present in areas in which people of higher socioeconomic status live [33,34].

### 4.2. Selection of Sampling Sites

We first identified green areas in the city using a vector layer of green areas available from INE [54]. Then, we selected public green areas larger than 60 m in width and length. To sample green areas of different sizes, we stratified them into three sizes: large (equal to or greater than 3 ha), medium (from 1.5 ha to 2.5 ha), and small (0.8 ha to 1 ha). Then, each green area was assigned the same socioeconomic level as the census block in which it is located using a digital layer of the socioeconomic distribution in the Santiago Metropolitan Area [55]. Socioeconomic levels can be divided into three main groups: high, medium, and low [56]. The high socioeconomic group is the wealthiest and most college educated, with an estimated average household income greater than USD 28,800 per year. The medium socioeconomic group is characterized by households with technical or high school education and an estimated average household income of over USD 13,200 per year. The low socioeconomic group is low income, has a lower educational level, and an estimated average household income of less than USD 8400 per year [57]. Finally, we used a stratified random selection approach to select 7 green areas in each combination of green area size (three levels: large, medium, and small) and socioeconomic level (three levels: high, medium, and low), leading to a potential sample size of 63 green areas. Due to the lack of a few large- and medium-sized green areas corresponding to the high socioeconomic level, 60 urban green areas were sampled (Figure 1). We note that areas with a high socioeconomic level only comprise 12.9% of the city, limiting the number of green areas available for sampling.

### 4.3. Tree Sampling

Sampling was conducted between September and October 2022. Trees were sampled in each green area using circular plots (11 m radius) [34]. The number of plots varied with green area size (small: 1 plot, medium: 2 plots, and large: 3 plots). In each plot, all the tree species present were identified. Trees with DBH > 2.5 cm were measured for height, DBH, and crown size (length and width). The crown size was used to calculate tree crown area (in m^2^). Then, tree cover was calculated by summing all tree crown areas within a plot.

### 4.4. Statistical Analysis

First, six variables were calculated per plot: total abundance and species richness, abundance and species richness of native trees, and abundance and species richness of introduced trees. To compare differences in the abundance and diversity of trees in green areas between socioeconomic groups, Generalized Linear Mixed Models (GLMMs) with Poisson distribution were fitted using the “lme4” package [58] in R software 4.3.1 [59]. Six models were fitted, each including one of the six response variables calculated per plot: total abundance and species richness, abundance and species richness of native trees, and abundance and species richness of introduced trees. All models included the socioeconomic level as a fixed effect (categorical variable with three levels: high, medium, low) and the green area as the random effect (n = 63).

Then, tree cover and DBH averages were compared among socioeconomic groups. For this, a Linear Mixed Effects Model (LMM) was used. Two models were constructed in which the response variables were tree cover and average DBH calculated per plot. All models included socioeconomic level as a fixed effect and green area as a random effect (n = 63).

Prior to interpreting the results of the models, all GLMMs with Poisson distribution were evaluated for overdispersion by calculating the sum of the square of the Pearson residuals and comparing it with the residual degrees of freedom of the model using chi-square tests [60]. Finally, in each model the effect of socioeconomic level was interpreted and then plotted using the “effects” package [61]. When the plot suggested differences between medium and low socioeconomic level, we performed Tukey’s tests for pairwise mean comparisons with the “multcomp” package [62].

## 5. Conclusions

No significant relationship was found between socioeconomic level and total tree abundance or tree species diversity in the green areas of Santiago de Chile. Although this suggests a lack of luxury effect in Santiago’s green areas, there was a differential effect according to the origin of the species. While introduced species in green areas did not exhibit a luxury effect, the richness of native species was higher in the high socioeconomic level areas than in the medium and low socioeconomic level areas. In addition, tree cover was higher in green areas located in high and medium socioeconomic level areas. Our results reveal differential access of people with different socioeconomic level to tree cover, to native species and, consequently, to the provision of ecosystem services and the possibility of knowing and interacting with the natural heritage in green areas of Santiago de Chile.

## Figures and Tables

**Table 1:** Number of plots and trees (native and introduced) recorded by socioeconomic level in green areas of Santiago de Chile.

	Total	Socioeconomic Level		
		High	Medium	Low
Number of green areas sampled	60	17	22	21
Number of plots sampled	117	32	43	42
Number of plots with trees	109	30	41	38
Number of trees	463	147	156	160
Number of native trees	141	60	34	47
Number of introduced trees	322	87	122	113
Number of trees/plot (mean ± SE)	3.96 ± 3.04	4.59 ± 3.40	3.63 ± 2.76	3.81 ± 2.95
Number of native trees/plot (mean ± SE)	1.21 ± 2.05	1.88 ± 2.63	0.79 ± 1.32	1.12 ± 2.04
Number of introduced trees/plot (mean ± SE)	2.75 ± 2.48	2.72 ± 2.67	2.84 ± 2.29	2.69 ± 2.51

**Table 2:** Abundance of trees (total and by origin) per hectare according to socioeconomic level in green areas of Santiago de Chile.

Socioeconomic Level	Number of Plots	Sampled Area (ha)	Total Tree Density (ind/ha)	Native Tree Density (ind/ha)	Introduced Tree Density (ind/ha)
High	32	1.22	120.85	49.32	71.52
Medium	43	1.63	95.44	20.8	74.64
Low	42	1.6	100.22	29.44	70.78

**Table 3:** Results of the Generalized Linear Mixed Model predicting the abundance and species richness of total, native, and introduced trees per plot in green areas within different socioeconomic levels in the city of Santiago de Chile.

Response Variable		Coefficient	Standard Error	*p*-Value
Total abundance	Intercept (Socioeconomic: High)	1.41	0.15	<0.001
	Socioeconomic: Medium	−0.27	0.20	0.19
	Socioeconomic: Low	−0.24	0.20	0.23
Total species richness	Intercept (Socioeconomic: High)	0.84	0.12	<0.001
	Socioeconomic: Medium	−0.08	0.16	0.62
	Socioeconomic: Low	−0.17	0.16	0.29
Abundance of native trees	Intercept (Socioeconomic: High)	−0.28	0.39	0.47
	Socioeconomic: Medium	−0.70	0.51	0.17
	Socioeconomic: Low	−0.50	0.51	0.33
Native tree species richness	Intercept (Socioeconomic: High)	−0.31	0.27	0.25
	Socioeconomic: Medium	−0.83	0.37	0.03
	Socioeconomic: Low	−0.59	0.35	0.09
Abundance of introduced trees	Intercept (Socioeconomic: High)	0.85	0.18	<0.001
	Socioeconomic: Medium	0.01	0.23	0.97
	Socioeconomic: Low	−0.06	0.23	0.79
Species richness of introduced trees	Intercept (Socioeconomic: High)	0.34	0.15	0.02
	Socioeconomic: Medium	0.23	0.19	0.22
	Socioeconomic: Low	0.05	0.20	0.80

**Table 4:** Results of the Linear Mixed Models predicting tree cover (m^2^) and average tree DBH per plot in green areas within different socioeconomic levels in the city of Santiago de Chile.

Response Variable		Coefficient	Standard Error	*p*-Value
Tree cover (m^2^)	Intercept (Socioeconomic: High)	174.63	27.19	<0.001
	Socioeconomic: Medium	−9.36	35.96	0.8
	Socioeconomic: Low	−63.72	36.17	0.08
Average tree DBH	Intercept (Socioeconomic: High)	20.23	3.35	<0.001
	Socioeconomic: Medium	7.85	4.44	0.08
	Socioeconomic: Low	−2.21	4.47	0.62

## Data Availability

Data will be available on request.

## Appendix A

**Table A1:** Abundance and origin of tree species recorded in 60 green areas of the city of Santiago de Chile. For species names, local and international catalogues of vascular plants were used [63,64,65,66,67,68,69,70]. For scientific names and authors, the International Plant Names Index and Plants of the World Online were used.

Local Common Name (Spanish)	Scientific Name and Author	Abundance	Relative Abundance (%)	Origin	Presence in Green Areas	Presence in Green Areas (%)
Espino	*Vachellia caven* (Molina) Seigler & Ebinger	49	10.6%	Native	5	8.3%
Quillay	*Quillaja saponaria* Molina	47	10.2%	Native	21	35.0%
Liquidámbar	*Liquidambar styraciflua* L.	35	7.6%	Introduced	13	21.7%
Ligustro	*Ligustrum lucidum* W.T.Aiton	22	4.8%	Introduced	6	10.0%
Jacarandá	*Jacaranda mimosifolia* D.Don	18	3.9%	Introduced	7	11.7%
Pimiento	*Schinus areira* L.	15	3.2%	Native	8	13.3%
Aromo australiano	*Acacia melanoxylon* R.Br.	13	2.8%	Introduced	4	6.7%
Tulipífero de Virginia	*Liriodendron tulipifera* L.	13	2.8%	Introduced	6	10.0%
Fresno norteño	*Fraxinus excelsior* L.	12	2.6%	Introduced	7	11.7%
Olmo común	*Ulmus minor* Mill.	12	2.6%	Introduced	4	6.7%
Maitén	*Maytenus boaria* Molina	11	2.4%	Native	6	10.0%
Acacia de tres espinas	*Gleditsia triacanthos* L.	10	2.2%	Introduced	7	11.7%
Roble español	*Quercus falcata* Michx.	10	2.2%	Introduced	6	10.0%
Árbol botella	*Brachychiton populneus* (Schott & Endl.) R.Br.	9	1.9%	Introduced	5	8.3%
Falso plátano	*Acer pseudoplatanus* L.	8	1.7%	Introduced	2	3.3%
Eucalipto rojo	*Eucalyptus camaldulensis* Dehnh.	8	1.7%	Introduced	1	1.7%
Nogal del Japón	*Ginkgo biloba* L.	8	1.7%	Introduced	5	8.3%
Árbol del paraíso	*Melia azedarach* L.	8	1.7%	Introduced	5	8.3%
Plátano híbrido	*Platanus × hispanica* Mill. ex Münchh.	8	1.7%	Introduced	4	6.7%
Pino australiano	*Casuarina cunninghamiana* Miq.	7	1.5%	Introduced	4	6.7%
Álamo negro	*Populus deltoides* W.Bartram ex Marshall	7	1.5%	Introduced	5	8.3%
Litre	*Lithraea caustica* (Molina) Hook. & Arn.	6	1.3%	Native	1	1.7%
Magnolia	*Magnolia grandiflora* L.	6	1.3%	Introduced	5	8.3%
Granada	*Punica granatum* L.	6	1.3%	Introduced	4	6.7%
Falsa acacia	*Robinia pseudoacacia* L.	6	1.3%	Introduced	3	5.0%
Pezuña de vaca	*Bauhinia forficata* Link	5	1.1%	Introduced	1	1.7%
Cedrus sp	*Cedrus sp.* Mill.	5	1.1%	Introduced	5	8.3%
Peumo	*Cryptocarya alba* (Molina) Looser	5	1.1%	Native	5	8.3%
Espinillo	*Parkinsonia aculeata* L.	5	1.1%	Introduced	2	3.3%
Sauce llorón	*Salix babylonica* L.	5	1.1%	Introduced	1	1.7%
Ciprés calvo	*Taxodium distichum* (L.) Rich.	5	1.1%	Introduced	3	5.0%
Pino bunya	*Araucaria bidwillii* Hook.	4	0.9%	Introduced	1	1.7%
Eucalipto blanco	*Eucalyptus globulus* Labill.	4	0.9%	Introduced	1	1.7%
Ciruelo rojo	*Prunus cerasifera* Ehrh.	4	0.9%	Introduced	4	6.7%
Roble albar	*Quercus robur* L.	4	0.9%	Introduced	3	5.0%
Alcornoque mediterráneo	*Quercus suber* L.	4	0.9%	Introduced	2	3.3%
Árbol de los dioses	*Ailanthus altissima* (Mill.) Swingle	3	0.6%	Introduced	2	3.3%
Lodón	*Celtis australis* L.	3	0.6%	Introduced	2	3.3%
Patagua	*Crinodendron patagua* Molina	3	0.6%	Native	1	1.7%
Ceibo de monte	*Erythrina falcata* Benth.	3	0.6%	Introduced	3	5.0%
Roble australiano	*Grevillea robusta* A.Cunn. ex R.Br.	3	0.6%	Introduced	3	5.0%
Árbol de Júpiter	*Lagerstroemia indica* L.	3	0.6%	Introduced	2	3.3%
Morera blanca	*Morus alba* L.	3	0.6%	Introduced	3	5.0%
Mayo	*Sophora macrocarpa* Sm.	3	0.6%	Native	1	1.7%
Acer negundo	*Acer negundo* L.	2	0.4%	Introduced	2	3.3%
Árbol de fuego	*Brachychiton acerifolius* (A.Cunn. ex G.Don) F.Muell.	2	0.4%	Introduced	1	1.7%
Olivo de Bohemia	*Elaeagnus angustifolia* L.	2	0.4%	Introduced	2	3.3%
Eucaliptus sp	*Eucalyptus sp.* L’Hér.	2	0.4%	Introduced	1	1.7%
Viscote negro	*Parasenegalia visco* (Lorentz ex Griseb.) Seigler & Ebinger	2	0.4%	Introduced	1	1.7%
Pinus sp	*Pinus sp.* L.	2	0.4%	Introduced	2	3.3%
Durazno	*Prunus persica* (L.) Batsch	2	0.4%	Introduced	1	1.7%
Tilo americano	*Tilia americana* L.	2	0.4%	Introduced	2	3.3%
Ulmus sp	*Ulmus sp.* L.	2	0.4%	Introduced	2	3.3%
Castaño de Indias	*Aesculus hippocastanum* L.	1	0.2%	Introduced	1	1.7%
Pino del Paraná	*Araucaria* angustifolia (Bertol.) Steud.	1	0.2%	Introduced	1	1.7%
Pewén	*Araucaria araucana* (Molina) K.Koch	1	0.2%	Native	1	1.7%
Casuarina sp	*Casuarina sp.* L.	1	0.2%	Introduced	1	1.7%
Árbol Indio	*Catalpa bignonioides* Walter	1	0.2%	Introduced	1	1.7%
Espino blanco	*Crataegus monogyna* Jacq.	1	0.2%	Introduced	1	1.7%
Ciprés llorón	*Cupressus funebris* Endl.	1	0.2%	Introduced	1	1.7%
Níspero japonés	*Eriobotrya japonica* (Thunb.) Lindl.	1	0.2%	Introduced	1	1.7%
Ceibo	*Erythrina crista-galli* L.	1	0.2%	Introduced	1	1.7%
Laurel	*Laurus nobilis* L.	1	0.2%	Introduced	1	1.7%
Mióporo	*Myoporum laetum* G.Forst.	1	0.2%	Introduced	1	1.7%
Roble bur	*Quercus macrocarpa* Endl.	1	0.2%	Introduced	1	1.7%
Quercus sp	*Quercus sp.* L.	1	0.2%	Introduced	1	1.7%
Molle	*Schinus latifolius* (Gillies ex Lindl.) Engl.	1	0.2%	Native	1	1.7%
Árbol de las pagodas	*Styphnolobium japonicum* (L.) Schott	1	0.2%	Introduced	1	1.7%
Olmo péndulo	*Ulmus glabra* Huds.	1	0.2%	Introduced	1	1.7%
Viburnum dulce	*Viburnum odoratissimum* Ker Gawl.	1	0.2%	Introduced	1	1.7%
